# Biallelic *CCM3* mutations cause a clonogenic survival advantage and endothelial cell stiffening

**DOI:** 10.1111/jcmm.14075

**Published:** 2018-12-13

**Authors:** Konrad Schwefel, Stefanie Spiegler, Sabine Ameling, Christiane D. Much, Robin A. Pilz, Oliver Otto, Uwe Völker, Ute Felbor, Matthias Rath

**Affiliations:** ^1^ Department of Human Genetics, University Medicine Greifswald and Interfaculty Institute for Genetics and Functional Genomics University of Greifswald Greifswald Germany; ^2^ Department of Functional Genomics Interfaculty Institute for Genetics and Functional Genomics, University Medicine Greifswald Greifswald Germany; ^3^ Centre for Innovation Competence ‐ Humoral Immune Reactions in Cardiovascular Diseases, University of Greifswald Greifswald Germany

**Keywords:** CCM3, cerebral cavernous malformations, CRISPR/Cas9 genome editing, endothelial cells, miRNA, real time deformability cytometry

## Abstract

CCM3, originally described as PDCD10, regulates blood‐brain barrier integrity and vascular maturation in vivo. *CCM3* loss‐of‐function variants predispose to cerebral cavernous malformations (CCM). Using CRISPR/Cas9 genome editing, we here present a model which mimics complete CCM3 inactivation in cavernous endothelial cells (ECs) of heterozygous mutation carriers. Notably, we established a viral‐ and plasmid‐free crRNA:tracrRNA:Cas9 ribonucleoprotein approach to introduce homozygous or compound heterozygous loss‐of‐function *CCM3* variants into human ECs and studied the molecular and functional effects of long‐term CCM3 inactivation. Induction of apoptosis, sprouting, migration, network and spheroid formation were significantly impaired upon prolonged CCM3 deficiency. Real‐time deformability cytometry demonstrated that loss of CCM3 induces profound changes in cell morphology and mechanics: CCM3‐deficient ECs have an increased cell area and elastic modulus. Small RNA profiling disclosed that CCM3 modulates the expression of miRNAs that are associated with endothelial ageing. In conclusion, the use of CRISPR/Cas9 genome editing provides new insight into the consequences of long‐term CCM3 inactivation in human ECs and supports the hypothesis that clonal expansion of CCM3‐deficient dysfunctional ECs contributes to CCM formation.

## 1 INTRODUCTION

Cerebral cavernous malformations (CCM) are clusters of thin‐walled, sinusoidal vascular channels within the brain or spinal cord. The inherited form of CCM is associated with heterozygous loss‐of‐function variants in either *CCM1* (*KRIT1*; OMIM: *604214), *CCM2* (*MGC4607*/*OSM*; *607929) or *CCM3* (*PDCD10*/*TFAR15*; *609118). A second somatic mutation within endothelial cells (ECs) is believed to initiate the pathogenesis of CCMs.^1^, ^2^ Leakiness or rupture of the fragile, low‐flow vascular structures can eventually lead to headaches, seizures or stroke. The prevalence of symptomatic hereditary CCM is 1:5400 to 1:6200.^3^ Notably, *CCM3* mutation carriers often present with an earlier age of onset and a higher bleeding risk than *CCM1* or *CCM2* mutation carriers.^4^, ^5^, ^6^

Little is known about the long‐term effects of CCM3 inactivation due to homozygous or compound heterozygous loss‐of‐function *CCM3* variants in human ECs. *CCM3* was initially described as an upregulated transcript in human TF**‐**1 erythroleukaemia cells upon GM‐CSF deprivation and was reported as CCM disease gene in 2005.^7^, ^8^ Pull‐down and co‐immunoprecipitation studies demonstrated that CCM3 forms a ternary complex with CCM1 and CCM2 in vitro and acts in intracellular networks with GCKIII serine/threonine kinases and other molecules.^9^ Its inactivation in ECs is associated with altered autophagy,^10^ impairment of the DLL4‐Notch pathway,^11^ activation of RhoA,^4^ MEKK3‐KLF2/4,^12^, ^13^, ^14^ BMP/TGF‐ß^15^ signalling and increased exocytosis of angiopoietin 2.^16^ Interestingly, the possibilities of the CRISPR/Cas9 system have not been used in these studies.

Since the clarification of the fundamental DNA interference mechanism and its first application for precise genome editing in mammalian cells,^17^, ^18^, ^19^ the CRISPR/Cas9 system has become a versatile research tool. However, its efficiency is cell type‐specific. Notably, CRISPR/Cas9‐mediated gene disruptions in umbilical vein ECs, microvascular ECs, coronary artery ECs or ECs derived from cord blood colony‐forming cells have almost exclusively been realized with viral delivery systems or plasmid transfections so far.^20^, ^21^, ^22^, ^23^, ^24^, ^25^, ^26^ While the occurrence of potential off‐target mutations may limit the use of both techniques, the delivery of the CRISPR/Cas9 components as ribonucleoprotein (RNP) complex can reduce off‐target effects due to its short intracellular half‐life.^27^ Yu and co‐workers have demonstrated that CRISPR/Cas9 RNPs can be used for genome editing in ECs.^28^ Its efficiency has been reported to be significantly lower than in easy‐to‐transfect cells though.

In this study, we used a crRNA:tracrRNA:Cas9 RNP‐based model of human cavernous malformations and siRNA transfections in parallel to study the molecular and functional effects of long‐term CCM3 deficiency in comparison to its acute knockdown. Hereby, we demonstrate that CCM3 inactivation in human ECs induces a clonogenic survival advantage of functionally impaired *CCM3* ^–/–^ ECs over time.

## 2 METHODS

### 2.1 Cell culture

Human umbilical vein endothelial cells (HUVEC; PromoCell, Heidelberg, Germany) and immortalized CI–huVECs (InSCREENeX, Braunschweig, Germany) were cultured in endothelial cell growth medium (ECGM; PromoCell) supplemented with 10% foetal calf serum (FCS, Thermo Fisher Scientific, Waltham, USA). Human cerebral microvascular endothelial cells (hCMEC/D3; Merck Millipore, Darmstadt, Germany) were cultured in EndoGRO‐MV complete medium (Merck Millipore) supplemented with 1 ng/mL FGF‐2 and 5% FCS. A limiting dilution assay was used to generate monoclonal cells. In brief, 15.000 cells/well were seeded in a 96‐well plate. After 24 hours, cells were serially diluted and seeded with statistically 0.5 cells/well in conditioned ECGM. Based on a previous literature report,^26^ the ROCK inhibitor Y–27632 (Enzo Life Sciences, Lausen, Switzerland) was supplemented once at seeding with 10 µmol/L to increase cloning efficiency. Clonal cells were first subcultured after 7‐14 days in ECGM with 10% FCS. If not stated otherwise, functional assays were carried out at passage 6 (HUVEC), 27‐31 (CI‐huVEC) and 31‐33 (hCMEC/D3). Acute *CCM3* and miR‐139‐5p inhibition as well as adenoviral‐mediated CCM3 re‐expression are described in Data S1.

### 2.2 CRISPR/Cas9 mediated in vitro mutagenesis

CRISPR/Cas9 target sequences located in exon 3 [5'**‐**CCT‐GTGTTTAATGAGGTGAGTTG**‐**3'; crRNA (e3)] and 6 [5'**‐**CCT‐AAACGAAAAGGCACGAGCAC‐3'; crRNA (e6)] of the *CCM3*gene [LRG_651 (LRG_651t1)] were identified with the CRISPRko algorithm (http://portals.broadinstitute.org/gpp/public/analysis-tools/sgrna-design) and E‐CRISP design tool (http://www.e-crisp.org/E-CRISP/). For RNP‐mediated genome editing, HUVECs, CI‐huVECs and hCMEC/D3 cells were transfected with crRNA:tracrRNA:Cas9 RNP‐complexes (Integrated DNA Technologies, Leuven, Belgium). In brief, 1 µmol/L crRNA:tracrRNA duplexes were complexed with 1 µmol/L *S.p.* Cas9 protein (IDT) in Opti‐MEM I reduced serum medium (Thermo Fisher Scientific) to a final concentration of 60 nmol/L. Transfection complexes were formed in Opti‐MEM with Lipofectamine RNAiMAX (Thermo Fisher Scientific). Subsequently, cells were reverse transfected with the RNP complexes and cultured in complete growth medium for at least 48 hours (final RNP concentration 10 nmol/L; 36 000 cells and 4.8 µl Lipofectamine RNAiMAX/well in 24‐well‐plates). Verification of genome editing efficiencies by amplicon deep sequencing and T7EI assays are described in Data S1.

### 2.3 RNA‐sequencing and quantitative PCR

Total RNA was extracted using PeqGold TriFast reagent (Peqlab‐VWR, Radnor, USA) and purified with Direct‐zol RNA MiniPrep Plus (Zymo Research, Irvine, USA) or Absolutely RNA miRNA Kit (Agilent Technologies, Santa Clara, USA). The NEXTflex Small RNA‐Seq kit v3 (Bioo Scientific, Austin, USA) was used for library preparation. Pooled libraries were sequenced with 1x51 cycles on a MiSeq instrument. Adapter sequences were trimmed with cutadapt v1.14 and the NOISeq package of the iSmaRT toolkit^29^ was used for read alignment and differential expression analysis. miRNA sequence data were uploaded to Gene Expression Omnibus (GEO) database (record number: GSE114947). Deregulated mature miRNAs were validated by qPCR using the qScript microRNA cDNA synthesis kit and the PerfeCTa SYBR Green SuperMix (Quantabio, Beverly, MA, USA) or LightCycler 480 SYBR Green I Master Mix (Roche, Mannheim, Germany) together with specific PerfeCTa microRNA Assays (Quantabio). For gene expression analysis, RNA was transcribed into cDNA with the First Strand cDNA Synthesis Kit (Thermo Fisher Scientific). The *CCM3* transcript levels were quantified on a QuantStudio 3D Digital PCR System (Thermo Fisher Scientific) with a *CCM3* specific Taqman assay (Hs00200578_m1, Thermo Fisher Scientific). Transcript expression of *CXCR4* was quantified by SYBR Green‐based qPCR on Roche Light Cycler 480 instrument II (Roche). The non‐coding RNAs *SNORD44* and *RNU6‐2* or the housekeeping genes *RPLP0* (ribosomal protein lateral stalk subunit P0) and *TBP* (TATA box binding protein, Hs00427620_m1) were used as endogenous controls, respectively.

### 2.4 Western blot

Proteins were extracted with PeqGold TriFast reagent (Peqlab‐VWR) and solubilized in buffer containing 8 M Urea, 2 M Thio‐Urea, and 20 mmol/L Tris for CCM3 immunoblotting. Total protein was separated on 10% TGX Stain‐Free FastCast SDS‐polyacrylamide gels (Bio‐Rad, Hercules, USA), transferred to PVDF‐membranes and immunostained with primary antibodies: rabbit anti‐CCM3 (1:300; IG‐626, ImmunoGlobe, Himmelstadt, Germany). An AP‐conjugated goat anti‐rabbit immunoglobulin secondary antibody (1:1000; D0487, Dako Denmark, Glostrup, Denmark) was used with BCIP/NBT chromogenic substrate for protein detection. A ChemiDoc XRS+ (Bio‐Rad) imager was used for gel/blot documentation of Stain‐Free total protein and colorimetric protein bands. Normalized band intensities were calculated with the ImageLab software (v6.0, Bio‐Rad) to semi‐quantify relative protein expression. Using total protein as loading control, the volume intensities of the detected protein bands were normalized to the volume intensities of the whole protein fraction. Chemiluminometric VEGFR2/pVEGFR2 detection is described in Data S1.

### 2.5 Real‐time deformability cytometry (RT‐DC) and immunofluorescence staining

RT‐DC analysis was performed as described before.^30^ Briefly, cells were centrifuged, resuspended in CellCarrier buffer (Zellmechanik Dresden, Dresden, Germany) to a concentration of 10^6^ cells/mL and analysed at three different flow rates. Cell size and deformation were used as analysis parameters, while an analytical model allowed to extract the elastic modulus to exclude size dependency of the CCM3 inactivation.^31^ For immunofluorescence analysis, cells were cultured on µ**‐**slides VI^0.4^ (Ibidi, Martinsried, Germany), fixed with 4% paraformaldehyde for 20 minutes, permeabilized with 0.1% Triton X‐100 for 15 minutes and blocked with 2% normal goat serum for 1 hour at room temperature. Primary antibodies against CD31 (1:50, mouse anti‐CD31, R&D, BBA7) and SM22α (1:350, rabbit anti‐SM22α, Abcam, ab14106) were visualized with conjugated secondary goat antibodies (1:200, anti‐mouse IgG Alexa Fluor 488, A**‐**11029 and 1:600, anti‐rabbit IgG Alexa Fluor 555, A‐21429, Life Technology). Cells were incubated at room temperature with primary and secondary antibodies for 1 hour each. DNA and F‐actin were stained with DAPI (Sigma‐Aldrich) and phalloidin (Abcam, Cambridge, UK), respectively.

### 2.6 Caspase‐3 activity and senescence

Caspase‐3 DEVD‐R110 Assay Kit (Biotium, Fremont, USA) was used for fluorometric analysis of Caspase‐3 activity according to the manufacturer's instructions. Endothelial apoptosis was induced with 0.25 or 1 µmol/L staurosporine for 2 hours. Cells were cultured for four days in endothelial cell basal medium (ECBM; PromoCell) containing 1% FCS before β‐galactosidase staining with the Cellular Senescence Kit (OZ Biosciences, San Diego, USA).

### 2.7 3D spheroid sprouting, migration, tube formation and proliferation assays

Spheroid formation and sprouting assay was performed as described previously.^32^ ImageJ software was used for data analysis. WST‐1 proliferation assay (Roche) was performed following manufacturer's instructions. For migration analyses, cell monolayers were scratched with a 200 µl pipet tip on a 24‐well plate. Images were made after 0 and 7 hours. The relative migrated area was calculated using ImageJ software. Tube formation was analysed using a Matrigel assay (Corning, Kaiserslautern, Germany). After gel matrix incubation at 37°C for 60 minutes, 20 000 cells/well were seeded in 96‐well plates. Tube formation was imaged after 18 hours and quantified with the angiogenesis analyzer for ImageJ (http://image.bio.methods.free.fr/ImageJ/).

### 2.8 Statistical analysis

Data were analysed with the GraphPad Prism software (v7.0a, GraphPad Software, La Jolla, USA) and presented as mean and standard deviation (SD) if not stated otherwise. Two‐tailed, one sample *t* tests were used for normalized data. Two or more groups were compared with a two‐tailed, Student's *t* test or one‐ and two‐way ANOVA, respectively. Dunnett or Šidák corrections were applied for multiple comparisons. False discovery rate (FDR; q) was used for analysis of small RNA‐sequencing data. Cell area and elastic moduli measurements were evaluated with the software ShapeOut (Zellmechanik Dresden) using linear mixed model comparisons. This approach allows to estimate the statistical significance of large datasets with multiple biological replicates. Briefly, linear mixed models separate RT‐DC data into two contributions — a fixed effect summarizing the impact of the knockout on cell mechanical properties and a random effect due to systematic or random measurement bias. After defining an additional model that lacks the fixed effect term a maximum likely hood ratio is calculated and the *P* value is derived. A *P* < 0.05 or q‐value <0.05 was regarded as statistically significant.

## 3 RESULTS

### 3.1 Efficient CRISPR/Cas9‐mediated *CCM3* gene disruption in human ECs

**Figure 1:**
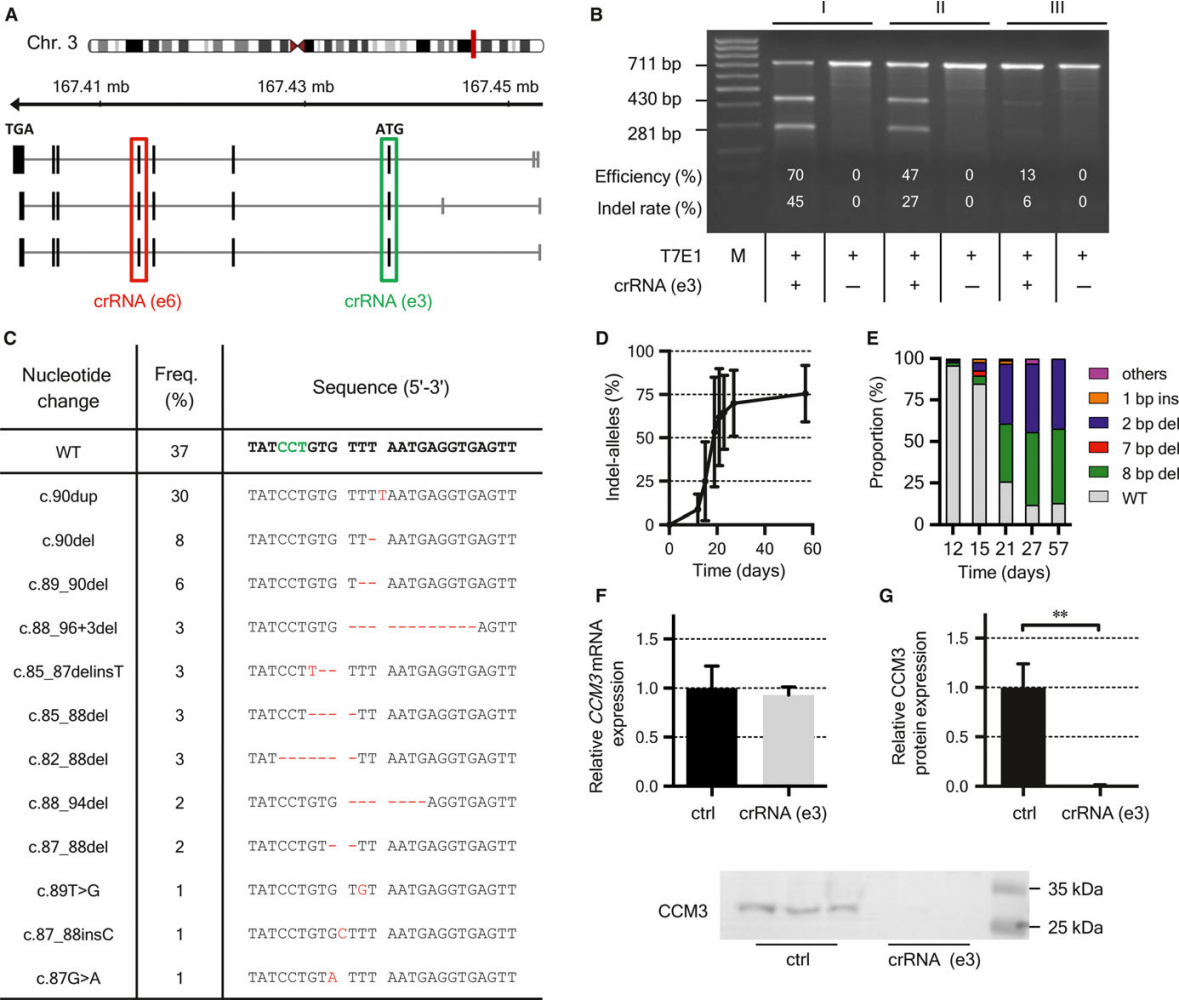
Efficient *CCM3* gene disruption in CI‐huVECs. A, The exon‐intron structure of the *CCM3* gene and its RefSeq transcripts are schematically depicted. CRISPR/Cas9 target sequences are highlighted in green and red. e = exon. B, Twelve days after crRNA(e3):tracrRNA:Cas9 transfection, T7EI assays indicated estimated indel rates of 6%‐45% in three independent replicates (I‐III). C, The mutational spectrum identified in a representative replicate 12 d after crRNA(e3):tracrRNA:Cas9 transfection is shown as sequence alignment. The PAM sequence is highlighted in green and nucleotide changes are marked in red. WT = reference allele. An increase of the total indel frequency (D) and a shift of the mutational spectrum (E) were observed after transfection. Mutations with variant frequencies ≤2% are summarized as “others”. Digital PCR revealed no difference in *CCM3* transcript expression (F) whereas no CCM3 protein was detectable 20‐23 d after crRNA(e3):tracrRNA:Cas9 transfection. Western blot results of three independent crRNA(e3):tracrRNA:Cas9‐treated cell pools are given in the lower panel (G). Data are presented as mean and SD (n = 3). ctrl = control. Two‐tailed, Student's *t* test was used for statistical analysis: **P < 0.01

To compare the efficiencies of viral and non‐viral CRISPR/Cas9 delivery systems for *CCM3* gene disruption in human ECs, we either transduced CI‐huVECs with lentiviral particles or transfected them with crRNA:tracrRNA:Cas9 RNPs. Locus‐specific effects were excluded since the same target sequence in the first coding exon of *CCM3* was used in both approaches [crRNA (e3); Figure 1A]. Fourteen days after lentiviral vector transduction and puromycin selection, *CCM3* mutant allele frequencies of 46%‐66% were detected by T7EI cleavage assay and amplicon deep sequencing (Figure S1). In the non‐viral approach, an average T7EI cleavage efficiency of 43% (range: 13%‐70%) was observed 12 days after RNP‐transfection without any selection for CRISPR/Cas9‐edited cells (Figure 1B). Amplicon deep sequencing demonstrated total *CCM3* mutant allele frequencies of up to 63% (Figure 1C). As expected for non‐homologous end‐joining repair, the majority of variants induced by viral and non‐viral CRISPR/Cas9 genome editing were small frameshift mutations located near the PAM site (±6 bp). The 1**‐**bp duplication c.90dupT was one of the predominant alleles whereas only very few deletions or insertions of more than 20 bp were identified (Figure 1C and Figure S1).

Notably, the frequency of crRNA:tracrRNA:Cas9 RNP‐induced *CCM3* mutant alleles significantly increased during culture time. Even in samples with low initial editing efficiencies, we observed *CCM3* indel frequencies of up to 88% after four to eight weeks (Figure 1D). Furthermore, we noticed a marked shift in the mutational spectrum of crRNA:tracrRNA:Cas9 RNP‐treated cells (Figure 1E). While *CCM3* mRNA expression was not reduced, CCM3 protein expression was completely abolished (Figure 1F,G). This observation is consistent with a previous CRISPR/Cas9 study targeting the *CIITA* locus in human ECs. Despite high genome editing efficiencies, no reduced *CIITA* transcript levels were found.^26^ Since off‐target effects are a concern in CRISPR/Cas9 genome editing experiments, we screened potential off‐target loci but found no CRISPR/Cas9‐induced variants (Table S1). In addition, we tested a second crRNA with a target sequence located in exon 6 which is shared by all *CCM3* transcript variants and encodes for a part of the FAT‐homology domain of CCM3. Increasing *CCM3* mutant allele frequencies were also found in this second approach. From day 12 to 18 after crRNA:tracrRNA:Cas9 RNP transfection, indel frequencies had more than doubled from 17% (range: 8%‐24%) to 38% (range: 27%‐48%).

To exclude cell‐type‐specific effects, we also used primary HUVECs and hCMEC/D3 cells. Consistent with our observations in CI‐huVECs, the relative number of *CCM3* indel alleles increased in both cell types after crRNA:tracrRNA:Cas9 RNP‐mediated genome editing (Figure S2).

### 3.2 Increased clonogenicity of *CCM3* ^–/–^ CI‐huVECs

**Figure 2:**
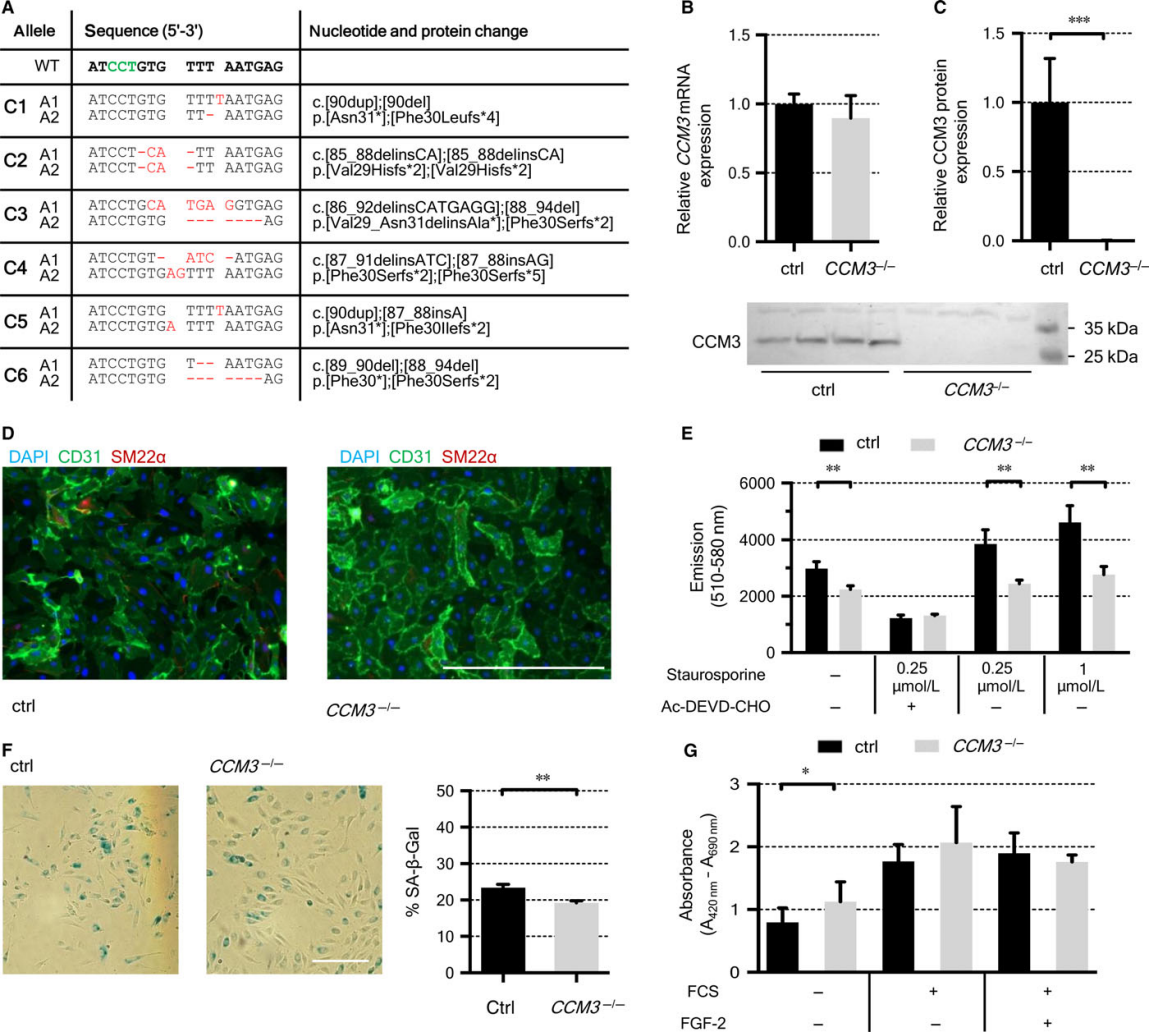
Characterization of clonally expanded *CCM3* ^–/–^CI–huVECs harbouring biallelic loss‐of‐function variants. A, Loss‐of‐function variants identified in six representative *CCM3* ^–/–^CI–huVEC clones (C1‐C6) are shown as sequence alignment. The PAM sequence is highlighted in green and nucleotide changes are marked in red. WT = reference allele. (B,C) *CCM3* mRNA expression was unchanged whereas no CCM3 protein was detected in *CCM3* ^–/–^CI**‐**huVECs. Western blot results of four *CCM3* ^–/–^and four control cell lines are given in the lower panel. (D) Immunofluorescence staining of CD31 (green), SM22α (red) and DAPI (blue) in wild‐type and *CCM3* ^–/–^ CI‐huVECs. Scale bar ≙ 400 µm. (E) *CCM3* ^–/–^CI**‐**huVECs demonstrated reduced Caspase‐3 activities under standard culture conditions and also upon staurosporine treatment. Ac‐DEVD‐CHO = synthetic inhibitor for Caspase‐3/7. F, Reduced senescence‐associated β‐galactosidase (SA‐β‐Gal) activity was observed in *CCM3* ^–/–^CI**‐**huVECs under serum starvation. Scale bar ≙ 200 µm. G, The proliferation rate of *CCM3* ^–/–^CI‐huVECs was slightly increased under serum starvation, whereas no difference was found in basal culture medium supplemented with 10% FCS and 25 ng/mL FGF‐2 or 10% FCS alone. Data are presented as mean and SD (n = 3‐8). Two‐tailed, Student's *t* test was used for statistical analysis: *P < 0.05; **P < 0.01; ***P < 0.001

To support the hypothesis that CCM3‐deficient ECs have a survival benefit, 58 CI‐huVEC clones were established by limiting dilution within three weeks. These were derived from two crRNA:tracrRNA:Cas9 RNP‐treated cultures which had *CCM3* mutant allele frequencies of 64% and 77%, respectively. Amplicon sequencing demonstrated compound heterozygosity for two distinct loss‐of‐function variants or homozygosity for a truncating *CCM3* mutation in 39 clones (67%) (Figure 2A). Of note, no cells with *CCM3* wild‐type alleles were found. All remaining 19 clones (33%) were compound heterozygous for a *CCM3* loss‐of‐function mutation and an in‐frame variant. Again, *CCM3* mRNA levels were not reduced in *CCM3* ^–/–^ CI‐huVECs with biallelic loss‐of‐function variants whereas CCM3 protein expression was completely abolished (Figure 2B,C). Only minor compensatory changes in *CCM1* and *CCM2* mRNA expression were observed (Figure S3). CCM3‐deficient CI**‐**huVECs maintained their endothelial identity with strong expression of platelet endothelial cell adhesion molecule‐1 (PECAM**‐**1/CD31) but low levels of smooth muscle protein 22‐alpha (SM22α) (Figure 2D).

The discrepancy between the observed and the expected number of *CCM3* wild‐type clones indicated a cell protective effect of CCM3 long‐term deficiency. In line with this observation, *CCM3* ^–/–^ CI‐huVECs demonstrated a significantly reduced activity of caspase 3 which is an indicator of apoptosis under standard culture conditions and also upon staurosporine treatment (Figure 2E). Moreover, senescence‐associated β‐galactosidase activity was slightly reduced in *CCM3* ^–/–^ CI‐huVECs under serum starvation (Figure 2F). Conversely, proliferation of serum‐starved *CCM3* ^–/–^ CI‐huVECs was increased (Figure 2G).

### 3.3 CCM3 modulates cell mechanics

**Figure 3:**
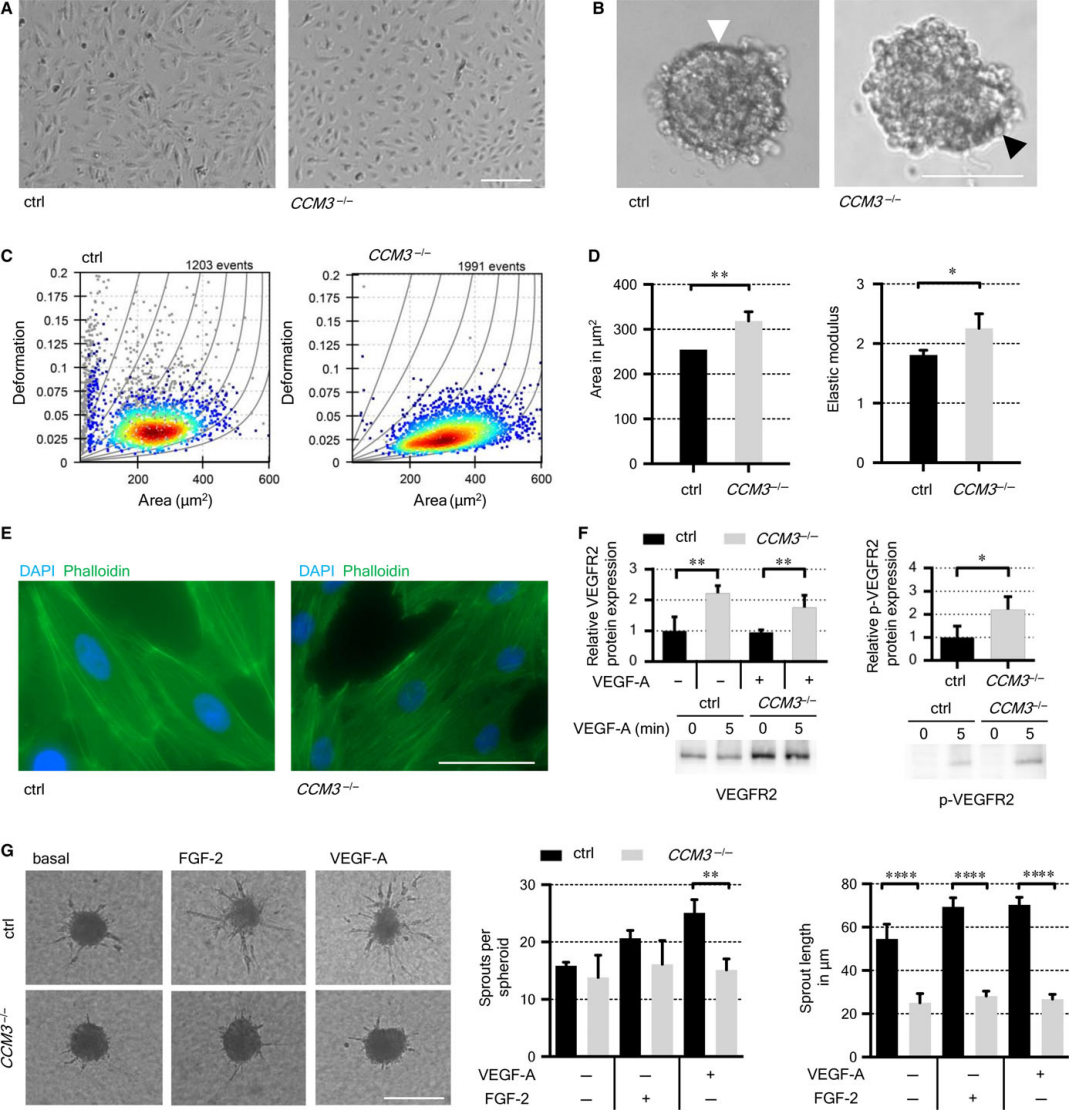
Changes of morphology, cytoskeleton and cellular function of *CCM3* ^–/–^CI**‐**huVECs. A, Representative bright‐field microscopy images indicated a more compact morphology of *CCM3* ^–/–^ CI‐huVECs. Scale bar ≙ 200 µm. B, Three‐dimensional spheroid organization of cells cultivated for 24 h in hanging drops. White arrowhead indicates properly formed spheroid surface monolayer while black arrowhead shows the irregular spheroid surface of CCM3‐deficient cells. Scale bar ≙ 100 µm. C, Representative dot plots of RT‐DC measurements for control and *CCM3* ^–/–^ CI‐huVECs. Deformation is depicted on the y‐axis and projected cell area on the x‐axis. D, An increased area and elastic modulus of *CCM3* ^–/–^ CI‐huVECs were observed in RT‐DC. E, Phalloidin‐iFluor 488‐(green) and DAPI‐(blue) staining demonstrated increased actin stress fibre content in *CCM3* ^–/–^ CI‐huVECs. Scale bar ≙ 50 µm. F, Protein level of VEGFR2 and its phosphorylated form (p‐VEGFR2) after VEGF‐A stimulation were significantly increased in *CCM3* ^–/–^ CI‐huVECs. Representative Western blot results of one *CCM3* ^–/–^ and one WT CI‐huVEC (ctrl) sample are shown in the lower panel. G, Spheroid sprouting assay demonstrated reduced sprout lengths of *CCM3* ^–/–^ CI‐huVECs under basal culture conditions and stimulation with 25 ng/mL VEGF‐A or FGF‐2. Scale bar ≙ 200 µm. ctrl = control. Data are presented as mean and SD (n = 3‐4). Student's *t* test, one sample *t* test, linear mixed models (RT‐DC) or two‐way ANOVA with Šidák's multiple comparisons test were used for statistical analyses: *P < 0.05; **P < 0.01; ****P < 0.0001

CCM3‐deficient CI‐huVECs demonstrated profound changes of endothelial cell shape, cell mechanics and cytoskeleton organization. Under adherent culture conditions, a more compact and rounded morphology was observed (Figure 3A). Furthermore, the ability to form round and clearly demarcated spheroids was markedly impaired in *CCM3* ^–/–^CI**‐**huVECs and all other analysed cell types upon long‐term CCM3 inactivation. When cultured as hanging drops, they aggregated but failed to properly organize and differentiate within 24 hours (Figure 3B and Figure S4). These observations were consistent with the results of our real‐time deformability cytometry assay which is a marker‐free way to characterize the mechanical phenotype of cells. RT‐DC demonstrated an increased cell area and elastic modulus of *CCM3* ^–/–^CI**‐**huVECs. The elastic modulus is independent of the cell surface area and its increase indicates a higher cell stiffness (Figure 3C, D).^30^ Since cell shape and cellular stiffness are critically linked to the endothelial cytoskeleton, actin filaments were visualized with phalloidin staining and immunofluorescence microscopy. While wild‐type cells demonstrated cortical actin localization, a reorganization was observed in *CCM3* ^–/–^CI**‐**huVECs which had more stress fibre bundles (Figure 3E).

Acute knockdown of CCM3 in HUVECs and CI‐huVECs by siRNA treatment also induced an increased actin stress fibre formation and cell stiffness but only clonal *CCM3* ^‐/‐^ CI‐huVECs demonstrated an increased cell area in RT‐DC (Figure S5 and data not shown). These results indicate that the reorganization of the actin cytoskeleton and the changes in cell mechanics are acute responses to CCM3 inactivation while changes of the endothelial cell shape are rather long‐term effects. Consistent with this hypothesis, a compact and rounded morphology was also observed in primary HUVECs upon crRNA:tracrRNA:Cas9 RNP‐induced long‐term CCM3 inactivation (Figure S2).

### 3.4 Reduced angiogenic response of *CCM3* ^–/–^ CI‐huVECs

**Figure 4:**
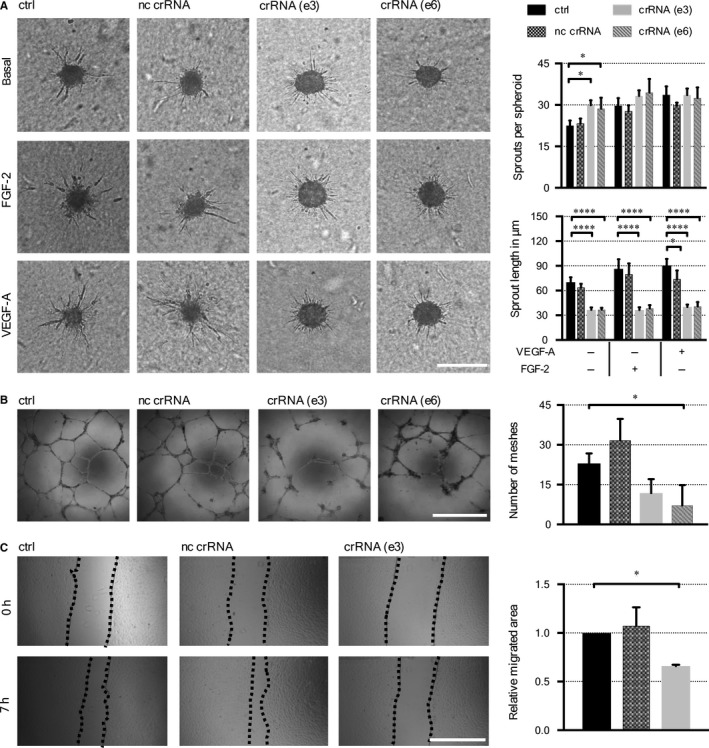
Reduced angiogenic response of CI‐huVECs after *CCM3* gene disruption. A, crRNA:tracrRNA:Cas9 RNP‐treated CI‐huVECs demonstrated reduced sprout lengths under basal culture conditions and stimulation with 25 ng/mL VEGF‐A or FGF‐2. Scale bar ≙ 200 µm. Decreased number of meshes in tube formation assay (B) and migration rates (C) were also observed. Scale bars ≙ 1 mm. nc crRNA = non‐targeting control crRNA, ctrl = untreated control. Data are presented as mean and SD (n = 3). One‐way or Two‐way ANOVA with Dunnett's multiple comparisons test and One‐sample *t* test were used for statistical analysis: *P < 0.05; ****P < 0.0001

Little is known about the long‐term effects of CCM3 deficiency in human ECs since most studies had focused on its acute inactivation so far.^11^, ^16^, ^33^ When we analysed the angiogenic properties of *CCM3* ^–/–^CI**‐**huVECs, we observed lower cell migration rates and significantly reduced endothelial sprouting although the expression of VEGFR2 and its phosphorylation after VEGF‐A treatment were upregulated twofold (Figure 3F,G and Figure S3). The elongation of endothelial sprouts was already reduced under basal culture conditions and did neither respond to VEGF‐A nor FGF‐2 treatment. Even excessive stimulation with 250 ng/mL VEGF‐A or FGF‐2 did not restore normal sprouting (data not shown). The effect of CCM3 long‐term deficiency on sprout number was less pronounced (Figure 3G). The reduced angiogenic properties were not restricted to clonal *CCM3* ^–/–^CI‐huVECs but were also observed in mixed crRNA:tracrRNA:Cas9 RNP‐treated CI‐huVECs that had not been cloned by limiting dilution. Upon genome editing of both *CCM3* target loci in CI‐huVECs [crRNA (e3) + (e6)], significantly impaired sprout elongation and migration were found (Figure 4A,C). While tube formation after 6 hours was unaffected (data not shown), the stability of tube‐like structures after 18 hours was significantly reduced in CCM3‐deficient CI**‐**huVECs (Figure 4B). This instability could not be rescued by short‐term *CCM3* re‐expression in *CCM3* ^–/–^CI**‐**huVECs (Figure S6). Of note, forced CCM3 expression in wild‐type CI‐huVECs also had an anti‐angiogenic effect when compared to cells transduced with the recombinant GFP control adenovirus (Figure S6). This result was not completely unexpected since it is well known that overexpression and knockout of genes encoding for multiprotein complex components can cause the same phenotype due to a negative impact on stoichiometry or interaction of the other binding partners.^34^ Furthermore, adenoviral transduction alone proved to be pro‐angiogenic, as it has been described in a previous report.^35^

Impaired sprouting and migration were only observed upon long‐term CCM3 inactivation. No such endothelial dysfunctions were found in CI‐huVECs from the same passage whose CCM3 expression had been transiently silenced by siRNA transfection (Figure S5). The chronic effects of CCM3 inactivation on the angiogenic phenotype of CI‐huVECs were also verified in primary HUVECs and hCMEC/D3 cells. Endothelial sprouting was significantly reduced in HUVECs that had been transduced with a lentiviral CRISPR/Cas9 vector and also in hCMEC/D3 cells upon crRNA:tracrRNA:Cas9 RNP‐induced *CCM3* gene disruption (Figure S7 and S8).

### 3.5 Altered expression pattern of ageing‐associated miRNAs in *CCM3* ^−/−^ECs

**Figure 5:**
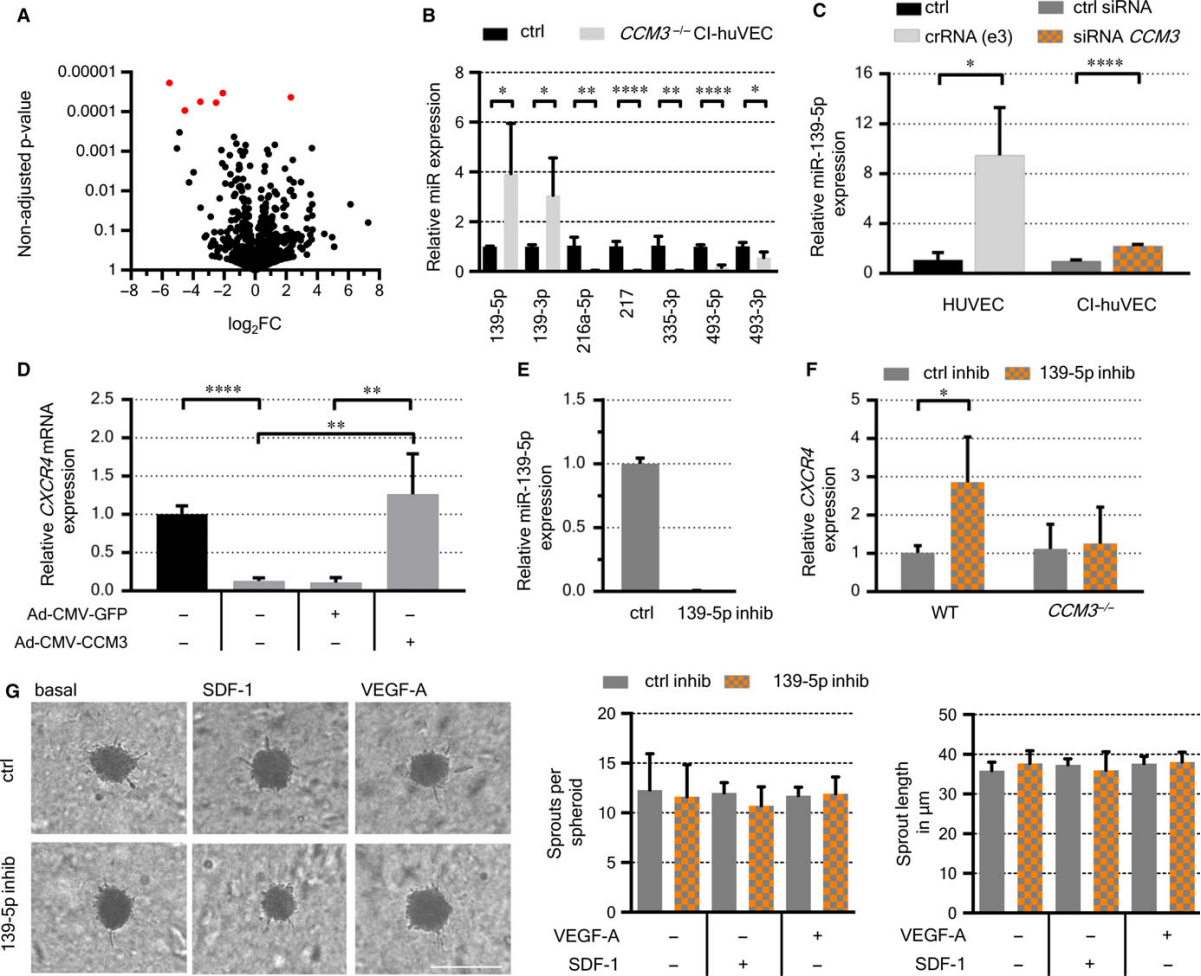
Deregulation of miR‐139‐5p and *CXCR4* in *CCM3* ^–/–^CI‐huVECs. A, Volcano plot of up‐ or downregulated mature miRNAs in *CCM3* ^–/–^CI‐huVECs. The log_2_ fold change (x‐axis) is plotted against the non‐adjusted *P*‐value (y‐axis). miRNAs with FDR‐adjusted q‐values <5% are marked in red. B, qPCR validation of the 3p and/or 5p strands of deregulated miRNAs found in *CCM3* ^–/–^CI‐huVECs. Ctrl = wild‐type CI‐huVECs. C, Relative miR‐139‐5p quantification in HUVECs (passage 18) after crRNA(e3):tracrRNA:Cas9 transfection and in CI‐huVEC following siRNA‐mediated CCM3 inactivation. D, *CXCR4*expression was significantly reduced in *CCM3* ^–/–^CI‐huVECs and could be restored by CCM3 re‐expression. E, Significant downregulation of miR‐139‐5p expression upon miR‐inhibitor transfection. F, Relative *CXCR4* mRNA expression after miR‐139‐5p inhibition in wild‐type (left) and *CCM3* ^–/–^CI‐huVECs (right). G, Sprouting was unaffected by acute miR‐139‐5p downregulation. Scale bar ≙ 200 µm. Data are presented as mean and SD (n = 3‐4). Two‐way ANOVA with Šidák's multiple comparisons test and Student's *t* test were used for statistical analysis: *P < 0.05; **P < 0.01; ****P < 0.0001

MicroRNAs (miRNAs) have become promising therapeutic targets in cancer, cardiovascular and infectious research.^36^ However, little is known about their role in CCM pathobiology. We therefore profiled the miRNA expression pattern of wild‐type and clonal *CCM3* ^–/–^ CI**‐**huVECs by small RNA sequencing. Of the 2588 mature miRNAs listed in the miRBase database v21, 439 and 452 were found to be expressed with mean RPM‐values ≥ 5 (reads per million reads) in wild‐type and *CCM3* ^–/–^CI**‐**huVECs, respectively. There was a large overlap with 402 miRNAs expressed in both conditions. When we checked for deregulated small RNAs in *CCM3* ^–/–^CI**‐**huVECs, the mature miRNAs hsa‐miR‐335‐3p (log_2 _FC = −5.52; q = 0.02), hsa‐miR‐217 (log_2 _FC = −4.53; q = 0.02), hsa‐miR‐216a‐3p (log_2 _FC = −3.54; q = 0.02), hsa‐miR‐493‐3p (log_2 _FC = −2.51; q = 0.02), hsa‐miR‐493‐5p (log_2 _FC = −2.08; q = 0.02) and hsa‐miR‐139‐3p (log_2 _FC = +2.29; q = 0.02) were found to be significantly up‐ or downregulated (Figure 5A). Since not only the mature miRNAs but in most cases also their corresponding precursor miRNAs (hsa‐mir‐493, hsa‐mir‐335, hsa‐mir‐216a, hsa‐mir‐217) were found to be regulated in the same direction, we considered miRNA biogenesis rather than miRNA degradation or turn‐over to be controlled by CCM3. Although the differences for the remaining corresponding strands did not pass the strict significance criteria, we also observed a trend towards decreased expression of hsa‐miR‐216a–5p (log_2 _FC = –4.87; q‐value = 0.06), hsa‐miR‐335‐5p (log_2 _FC = −5.05; q**‐**value = 0.07) and higher levels of hsa‐miR‐139‐5p (log_2 _FC = +2.42; q = 0.09). Expression differences were verified by RT‐qPCR analysis with miR‐139‐5p being upregulated nearly 4‐fold (Figure 5B).

Prediction of miRNA‐target interactions and pathway analysis with the miRNet algorithm^37^ demonstrated an enrichment of various gene ontology gene sets. Notably, “vasculature development” (*P* = 0.006) and “ageing” (*P* = 0.007) were among the most significantly enriched gene ontology biological process terms. For example, the miR‐217 level that has previously been reported as upregulated in senescent ECs^38^ was found to be reduced by more than 95% in *CCM3* ^–/–^CI**‐**huVECs when compared to wild‐type cells (129 vs. 2989 RPM). Since ageing appears to be a major biological process influenced by CCM3 deficiency, we analysed the expression level of the identified miRNAs in high‐passage, CRISPR/Cas9 RNP‐treated and wild‐type HUVECs and found miR‐139‐5p and miR‐139‐3p to be significantly upregulated in CCM3‐deficient HUVECs (Figure 5C and Figure S9).

### 3.6 miR‐139‐5p inhibition is not a treatment option for CCM3 deficiency

The observation that miR‐139‐5p was also upregulated more than twofold in CI**‐**huVECs upon acute CCM3 inactivation prompted us to address its function in CCM3‐deficient ECs in more detail (Figure 5C). Since the proangiogenic CXC chemokine receptor 4 (*CXCR4*) gene is a validated target of miR‐139‐5p,^39^ we first analysed its expression in CI‐huVECs upon long‐term CCM3 inactivation. As expected, *CXCR4* transcript levels were significantly reduced in *CCM3* ^–/–^CI**‐**huVECs. Because re‐expression of wild‐type CCM3 restored *CXCR4* levels, a regulatory CCM3/CXCR4 axis was assumed (Figure 5D). In order to further delineate the role of miR‐139‐5p in this axis, we silenced its signalling by transfection of a specific miRNA inhibitor (Figure 5E). While negative regulation of *CXCR4* by miR‐139‐5p was intact in wild‐type CI‐huVECs, it was compensated in *CCM3* ^–/–^ECs (Figure 5F). In agreement with this observation, neither apoptosis induction nor sprouting, endothelial network formation or migration could be restored by miR‐139‐5p inhibition (Figure 5G and Figure S9).

## 4 DISCUSSION

The lack of approved CCM therapies highlights that our understanding of the signalling cascades by which CCM3 controls endothelial quiescence and supports blood‐brain barrier (BBB) integrity is still incomplete. For the first time, we have therefore used the CRISPR/Cas9 technology to generate an in vitro knockout model which mimics complete CCM3 inactivation by a second somatic mutation in heterozygous mutation carriers.^1^, ^2^ Using the non‐viral and selection‐free CRISPR/Cas9 RNP delivery approach, we observed *CCM3* indel frequencies in human ECs that were significantly higher than those described for other target genes so far.^28^ Thus, we have generated a model which enabled us to address the molecular and functional effects of chronic CCM3 inactivation in human ECs. Our results illustrate that crRNA:tracrRNA:Cas9 RNPs, which minimize the risk of off‐target effects, can be used as a powerful and straightforward tool for genome editing in ECs.

With NGS‐based tracking of *CCM3* mutant allele frequencies after genome editing and use of a limiting dilution cloning assay, we have clearly shown that *CCM3* ^–/–^ECs have a clonal survival advantage. In particular, activation of the caspase 3 apoptotic cascade was significantly impaired in *CCM3* ^–/–^CI**‐**huVEC clones that harbour biallelic loss‐of‐function variants. The effects of CCM3 on cell death can be variable in different cell types and micro‐environmental conditions but our observations emphasize that CCM3 modulates apoptosis not only in serum‐starved or staurosporine‐treated cells but also in non‐stressed ECs.^40^, ^41^, ^42^, ^43^, ^44^, ^45^ Of note, wild‐type CI‐huVECs displayed signs of replicative senescence and growth arrest upon long‐term culture. In contrast, *CCM3* ^–/–^CI**‐**huVEC clones proliferated well to high passages and had slightly lower SA‐β‐gal activities under serum starvation which is in agreement with observations from *CCM3*‐silenced primary human coronary artery ECs after replicative stress.^46^ Taken together, these results suggest that clonal expansion of mutant ECs contributes to CCM pathobiology.^47^

Since CCM3 facilitates activation of pro‐apoptotic cascades in ECs, it is reasonable to conclude that its chronic inactivation counteracts the removal of dysfunctional *CCM3* ^–/–^ECs. Consequently, not only acute but particularly long‐term effects of CCM3 depletion become important. Using CRISPR/Cas9 genome editing and RNAi in parallel, we have demonstrated for the first time that ECs become larger and less deformable upon chronic CCM3 deficiency. Cellular stiffening is known to cause destabilization of cell‐cell junctions and to promote vascular permeability.^48^ As a logical consequence, the ability of *CCM3* ^–/–^ECs to form organized spheroids in 3D culture was found to be markedly impaired.

We also demonstrated that chronic CCM3 inactivation compromises the angiogenic properties of ECs whereas its acute silencing does not. Notably, increased migration and sprouting,^11^, ^16^, ^33^ but also impaired endothelial network formation of *CCM3*‐silenced ECs have been reported.^49^, ^50^, ^51^ Upon chronic CCM3 ablation in human ECs, we observed dramatically reduced endothelial sprouting and migration despite strong activation of VEGFR2 signalling. However, the impact of this signalling cascade on CCM biology remains controversial. CCM3 inactivation has been shown to block VEGF signalling by destabilization of VEGFR2 in vitro and in vivo.^50^ On the contrary, increased VEGFR2 activation and mRNA expression have been observed in human pulmonary artery ECs upon CCM1 inactivation and in HUVECs upon *CCM3* silencing, respectively.^11^, ^52^ The discrepancy between the activation status of *CCM3* ^–/–^CI‐huVECs and their in vitro angiogenic behaviour indicates that chronic CCM3 inactivation triggers compensatory mechanisms. This hypothesis is in line with the observation that different pathways become sequentially activated after *Ccm3* ablation in ECs of *Ccm3* ^ECKO^mice.^53^

miRNAs can orchestrate complex endothelial networks and we hypothesized that these small non‐coding RNAs might be part of the adaptive response of ECs to chronic CCM3 depletion. We therefore profiled the miRNA expression pattern of *CCM3* ^–/–^CI‐huVECs and demonstrated for the first time that CCM3 regulates the expression of miRNAs that are associated with ageing and vascular development. miR‐216a and miR‐217 have been reported to be upregulated during endothelial ageing. Notably, forced expression of miR‐217 and miR‐216a induces premature endothelial senescence.^38^, ^54^ Their strong downregulation in *CCM3* ^–/–^ CI**‐**huVECs fits well to the higher replicative potential of CCM3‐deficient ECs.

Another deregulated miRNA, miR‐139‐5p, which was significantly upregulated upon acute and chronic CCM3 inactivation in CI‐huVECs is known to control *CXCR4* gene expression on a posttranscriptional level.^39^ CXCR4 is a tip cell‐enriched receptor whose activation promotes endothelial migration and filopodia development.^39^, ^55^ It is also a key component of Notch‐controlled sprouting angiogenesis and needs to be tightly regulated for proper vascular maturation.^56^, ^57^ Notably, its expression is not only reduced in *CCM3* ^–/–^ CI**‐**huVECs but also in brain microvascular ECs of *Krit1* ^ECKO^ mice.^58^ Rescue of *CXCR4* levels by adenoviral CCM3 re‐expression in *CCM3* ^–/–^ CI‐huVECs indicates that CCM3 positively regulates *CXCR4* expression. Our study therefore represents an independent experimental approach suggesting that *CXCR4* downregulation might be a common consequence of CCM1 and CCM3 inactivation. However, this regulatory axis is only partially controlled by miR‐139‐5p. While negative regulation of *CXCR4* by miR‐139‐5p was validated in wild‐type CI**‐**huVECs, it was found to be compensated after chronic CCM3 inactivation. Consequently, neither *CXCR4* expression nor the dysfunctional angiogenic properties of *CCM3* ^–/–^CI**‐**huVECs could be rescued by miR‐139‐5p inhibition alone. Taken together, these results support the hypothesis that miR‐139‐5p may modulate phenotypic variability^59^ but is not a promising therapeutic target within CCM pathogenesis.

In conclusion, our genome editing study demonstrates that long‐term CCM3 inactivation rather than its acute knockdown induces a clonogenic survival benefit and disturbances of endothelial network formation and angiogenic properties. This novel in vitro model may be useful to identify new therapeutic targets to block clonal expansion of mutant ECs upon chronic CCM3 depletion.

## CONFLICT OF INTEREST

OO is shareholder in a company distributing real‐time deformability cytometry.

## AUTHOR CONTRIBUTIONS

KS, SSp, CDM, SA and RAP performed most of the experiments. MR, UV and UF contributed to the intellectual conception and the design of the study and supervised the experiments. KS, SSp, CDM, SA and MR analysed the data. OO performed and analysed all real‐time deformability cytometry experiments. All authors contributed to interpretation of the results. MR, KS and UF drafted the manuscript and all authors contributed to writing.
