# Supplementary material for: Biallelic CCM3 mutations cause a clonogenic survival advantage and endothelial cell stiffening
Source: J Cell Mol Med. 2018 Dec 13;23(3):1771–83. doi: 10.1111/jcmm.14075 (PMC6378188; doi:10.1111/jcmm.14075)
Supplement: Supplementary file 1 [file JCMM-23-1771-s001.pdf]

Supporting Information

Biallelic *CCM3* mutations cause a clonogenic survival advantage and endothelial cell stiffening

Konrad Schwefel[1], Stefanie Spiegler[1], Sabine Ameling[2], Christiane D. Much[1], Robin A. Pilz[1], Oliver Otto[3], Uwe Völker[2], Ute Felbor[1], Matthias Rath[1,*]

[1] Interfaculty Institute for Genetics and Functional Genomics, University of Greifswald and Department of Human Genetics, University Medicine Greifswald, Greifswald, Germany

[2] Interfaculty Institute for Genetics and Functional Genomics, Department of Functional Genomics, University Medicine Greifswald, Greifswald, Germany

[3] Centre for Innovation Competence - Humoral Immune Reactions in Cardiovascular Diseases, University of Greifswald, Greifswald, Germany

Corresponding Author*

Matthias Rath, MD
University Medicine Greifswald - Department of Human Genetics
Fleischmannstraße 43
D-17475 Greifswald
Germany
E-Mail: rathm@uni-greifswald.de
Phone: +49-3834-86-5396
Fax: +49-3834-86-5369

Supplementary Methods:

Lentiviral CRISPR/Cas delivery

LentiCRISPR v2 (Addgene plasmid #52961) was used for lentiviral CRISPR/Cas9 genome editing following established protocols [1]. Lentiviral particles were produced as described previously [2]. Briefly, psPAX2 (0.9 µg; Addgene #12260), pCMV-VSV-G (0.1 µg; #8454), and lentiCRISPR v2 (1 µg) were transfected into HEK293T cells on 6 cm culture dishes at 70% confluency using Lipofectamine 2000 (Thermo Fisher Scientific). Plasmids were kind gifts from Didier Trono, Bob Weinberg and Feng Zhang. The medium was replaced with DMEM (Thermo Fisher Scientific) supplemented with 30% FCS after 18 h. Supernatants were collected 24 h, 48 h and 72 h later. After concentration with Lenti-X-Concentrator (Clontech, Mountain View, USA), viral titers were determined. CI-huVECs and HUVECs were infected with lentiviral particles using ECGM containing 10% FCS and 8 µg/ml Polybrene (Sigma-Aldrich, St. Louis, USA). ECGM with 10% FCS and 1,5 µg/ml Puromycin (Sigma-Aldrich) was used for selection. Lentiviral titers were determined by serial dilution of lentiviral particles. 14 days after transduction, the transforming units per milliliter were calculated by staining colonies with crystal violet solution (Sigma-Aldrich).

T7EI cleavage assay and amplicon deep sequencing

Genome editing efficiencies were determined by digesting annealed PCR amplicons with T7EI (New England Biolabs, Frankfurt am Main, Germany). Fragments were visualized by gel electrophoresis. Estimated indel frequencies were calculated as described before [3]. The CRISPR/Cas9-induced mutational spectrum was analyzed by amplicon deep sequencing. Libraries were prepared in a custom two-step PCR approach. PCR products were purified with Agencourt AMPure XP beads (Beckman Coulter, Pasadena, USA), pooled and sequenced on a MiSeq instrument with 2x150 cycles (Illumina, San Diego, USA). The SeqNext software was used for data analysis (JSI Medical Systems, Ettenheim, Germany). Only variants with combined read frequencies $\geq$ 1% and quality score $\geq$ 25 were called. Potential off-target loci were identified with CCTop (https://crispr.cos.uni-heidelberg.de/).

Transient knockdown and re-expression studies

CCM3 expression was transiently knocked down by transfection of a *CCM3*-specific siRNA (Silencer select siRNA ID: s22176, Thermo Fisher Scientific) following established protocols [4]. A non-targeting siRNA was used as control. miR-139-5p signalling was transiently silenced by transfection with 50 nM of a specific miRNA inhibitor (LightSwitch ID: INH0170, Active Motif, La Hulpe, Belgium) using Lipofectamine RNAiMAX (Thermo Fisher Scientific). Inhibitor negative control v1 (Active Motif) was used as control. For CCM3 re-expression, DNA coding for human *CCM3* was introduced into CI-huVECs using adenoviral particles (Vector Biolabs, Malvern, USA, #ADV-218433). Transduction was performed according to the manufacturer's instructions with an MOI of 50. A recombinant Ad-CMV-GFP (Vector Biolabs, #1060) was used as control.

Western Blot analysis VEGFR2/p-VEGFR2

RIPA buffer containing Halt™ protease and phosphatase inhibitor cocktail (Thermo Fisher Scientific) was used to lyse cells for VEGFR2/p-VEGFR2 quantification after stimulation with 10ng/ml VEGF-A for 5min. Protein separation, transfer, documentation and data analysis were done as described in the main text. Rabbit anti-VEGFR2 (1:1,000; #2479, Cell Signaling, Frankfurt, Germany), rabbit anti-p-VEGFR2 (Tyr1175) (1:500; #2478, Cell Signaling) were used for immunostaining. A HRP-conjugated secondary antibody was used for chemiluminometric detection: mouse anti-rabbit (1:5,000; sc-2357, Santa Cruz).

Supplementary Figures:

nucleotide change	frequency (%)	sequence (5'-3')
WT	34	**TATCCTGTG TTT AATGAGGTGAGTT**
c.90dup	20	TATCCTGTG TTTTAATGAGGTGAGTT
c.88_96+3del	11	TATCCTGTG --- ---------AGTT
c.85_88del	9	TATCCT--- -TT AATGAGGTGAGTT
c.87_88insAG	8	TATCCTGTGAGTTT AATGAGGTGAGTT
c.88_96+5delinsATA	6	TATCCTGTGA TA- ----------TT
c.90del	4	TATCCTGTG TT- AATGAGGTGAGTT
c.88T>A	2	TATCCTGTA ATT AATGAGGTGAGTT
c.82_89delinsGTGAGTTGGG	2	TATGTGAGTTGGGT AATGAGGTGAGTT
c.82_93del	2	TAT------ --- ---GAGGTGAGTT
c.87_88ins§	2	TATCCTGTG§ TTT AATGAGGTGAGTT

§ ACTAAATTAAAATGGTGATTAAAATGTATCACCAT

Figure S1. Lentiviral *CCM3* gene disruption in CI-huVECs. Mutational signature of CI-huVECs transduced with lentiviral particles for CRISPR/Cas9 mediated editing of *CCM3* exon 3. The PAM sequence is highlighted in green and nucleotide changes are marked in red. WT = reference allele.

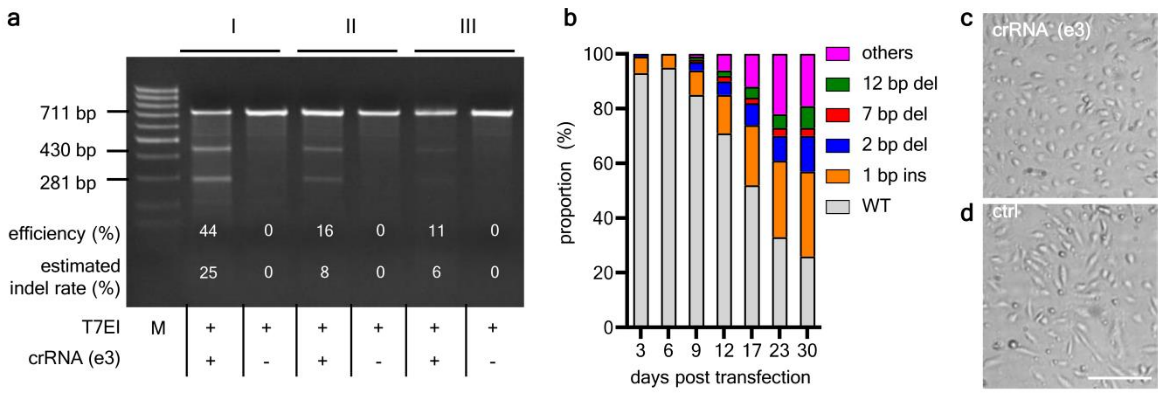

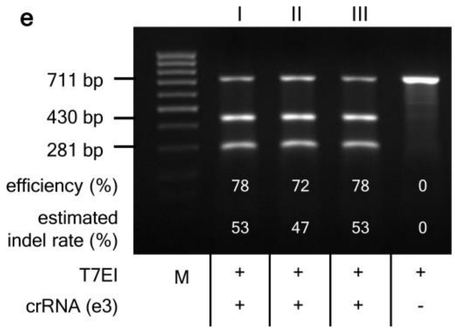

days after transfection	11		21	
T7E1 cleavage	efficiency (%)	estimated indel rate (%)	efficiency (%)	estimated indel rate (%)
exon 3 (N=3)	76	51	78	53
exon 6 (N=3)	46	26	63	39

Figure S2. Efficient CRISPR/Cas9-RNP-induced *CCM3* gene disruption in HUVECs and hCMEC/D3 cells. (a) T7EI assay indicated estimated indel frequencies of 6 to 25% in HUVECs 72 h after crRNA(e3):tracrRNA:Cas9 transfection. Three independent replicates (I-III) are shown. (b) A shift of the CRISPR/Cas9-induced mutational spectrum was observed in amplicon deep sequencing over time. One representative replicate is shown. Distinct mutations with variant frequencies $\leq 2\%$ are summarized as “others”. WT = reference allele. (c,d) A more compact morphology was observed in bright field microscopy for crRNA(e3):tracrRNA:Cas9-treated HUVECs. ctrl = control. Scale bar ≙ 200 µm. (e) High estimated indel frequencies and an increase of indel alleles during overall culture time were also observed in hCMEC/D3 cells upon crRNA:tracrRNA:Cas9 transfection.

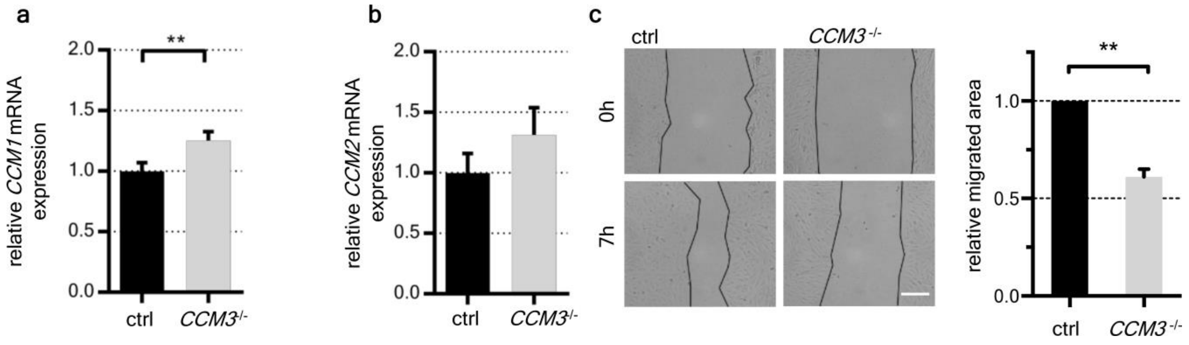

Figure S3. ***CCM1*** **(a) and** ***CCM2*** **(b) expression in and migration (c) of** ***CCM3*****$^{-/-}$ CI-huVECs.** Data are presented as mean and SD (n = 3-4). Student's t-test and One-sample t-test were used for statistical analysis: ** p<0.01.

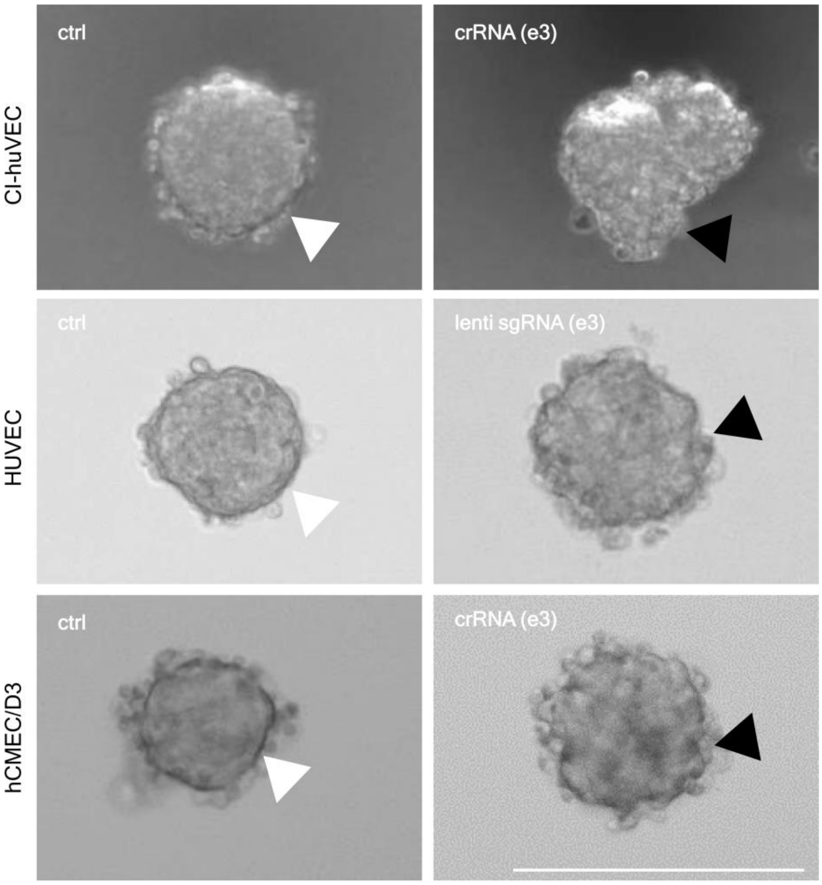

Figure S4. Endothelial cells with CCM3-deficiency fail to organize properly in three-dimensional spheroids. Three-dimensional spheroid organization of endothelial cells cultivated for 24 h in hanging drops. ctrl = control. Scale bar ≙ 200 µm. White arrowheads indicate the properly formed spheroid surface monolayer of control cells while black arrowheads show the unevenly formed spheroid surface of CCM3-deficient cells.

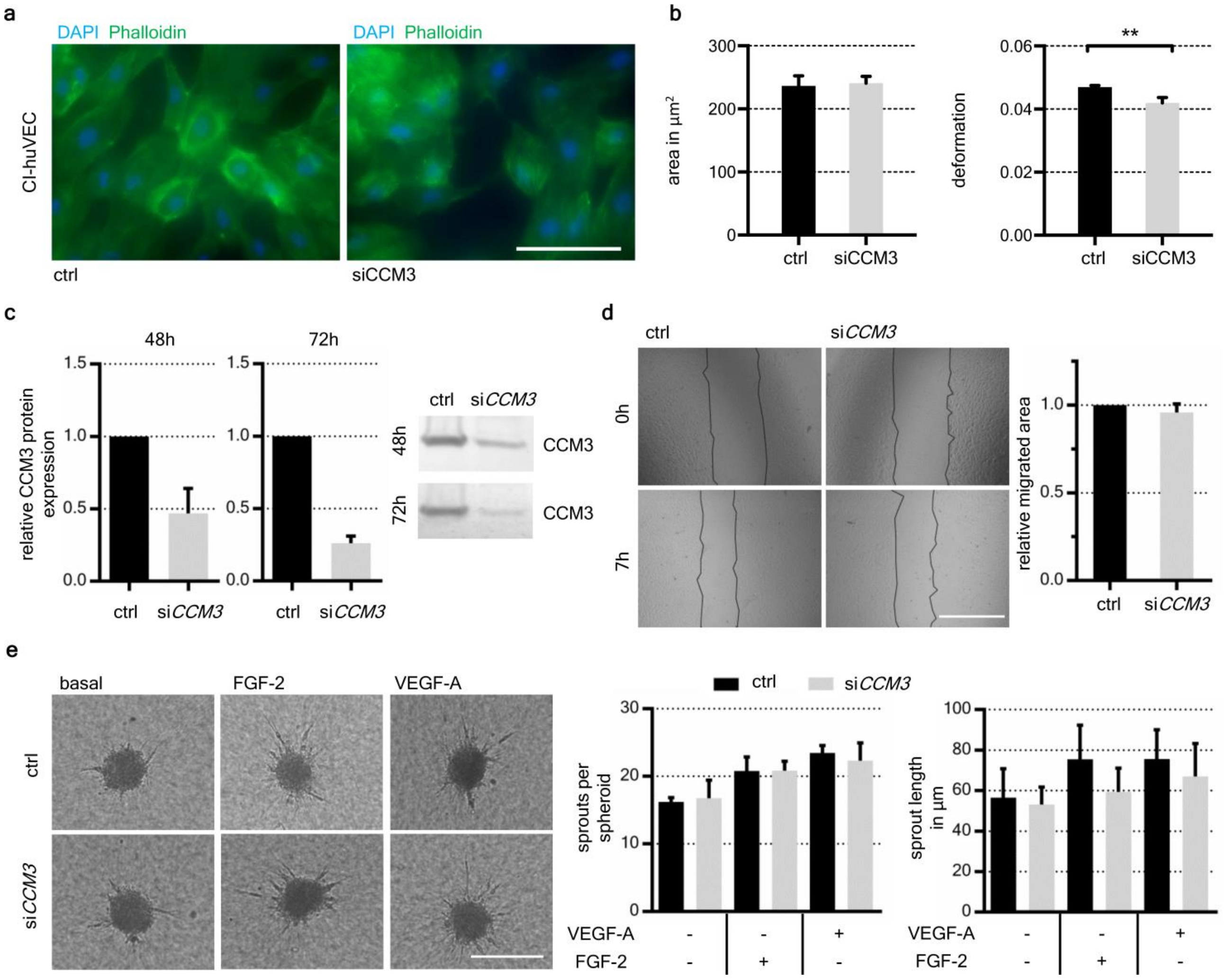

Figure S5. Acute CCM3 inactivation in CI-huVECs. (a) Immunofluorescence staining of the actin cytoskeleton (Phalloidin-iFluor 488) in CI-huVECs 48 h after transient *CCM3* knockdown. DAPI (blue) was used to counterstain DNA. (b) Cell area and deformability were measured by RT-DC. (c) Down-regulation of CCM3 protein expression 48 and 72 h after siRNA transfection. Migration (d) and sprouting (e) of CI-huVECs were unaffected by acute CCM3 silencing. Scale bars ≙ 100 µm (a), 1 mm (d) or 200 µm (e). ctrl = non-targeting siRNA. Data are presented as mean and SD (n = 3). Linear mixed models (RT-DC), Two-way ANOVA with Šidák's multiple comparisons test and One-sample t-test were used for statistical analysis: ** p<0.01.

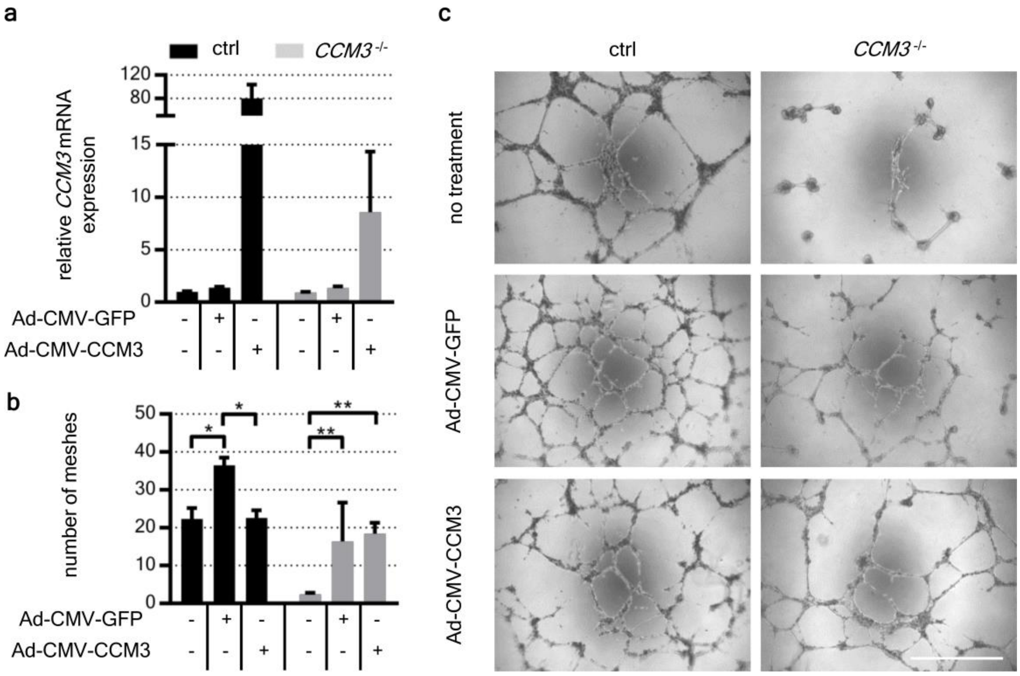

Figure S6. *CCM3* re-expression in *CCM3*$^{-/-}$ CI-huVECs. (a) Relative *CCM3* mRNA expression 72 h after adenoviral transduction. (b-c) Tube formation analysis revealed a pro-angiogenic effect of adenoviral infection alone, but no rescue of endothelial network formation after acute *CCM3* re-expression in comparison to the GFP control. Scale bar ≙ 1mm. ctrl = wild-type CI-huVECs. Data are presented as mean and SD (n=3-4). Two-way ANOVA with Šidák's multiple comparisons test was used for statistical analysis. * $p<0.05$; ** $p<0.01$.

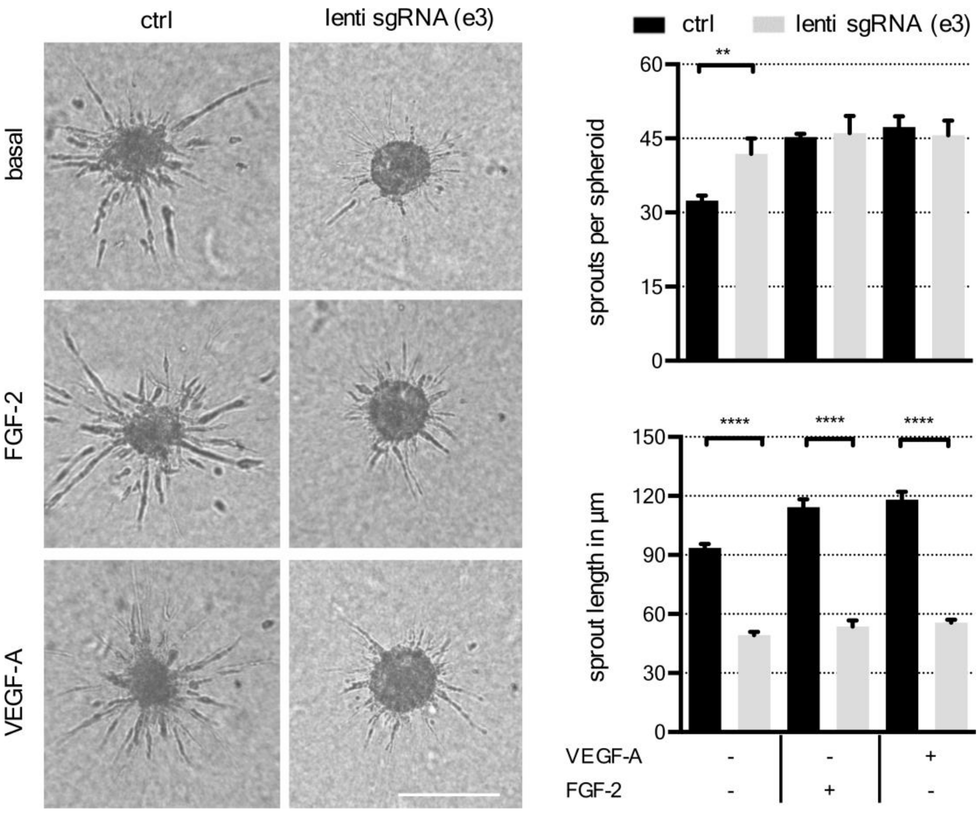

Figure S7. Reduced angiogenic response of HUVECs after lentiviral *CCM3* gene disruption. Sprout lengths were significantly reduced in HUVECs after lentiviral *CCM3* gene disruption. Scale bar ≙ 200 µm. ctrl = control. Data are presented as mean and SD (n = 3). Two-way ANOVA with Šidák's multiple comparisons test was used for statistical analysis: ** p<0.01; **** p<0.0001.

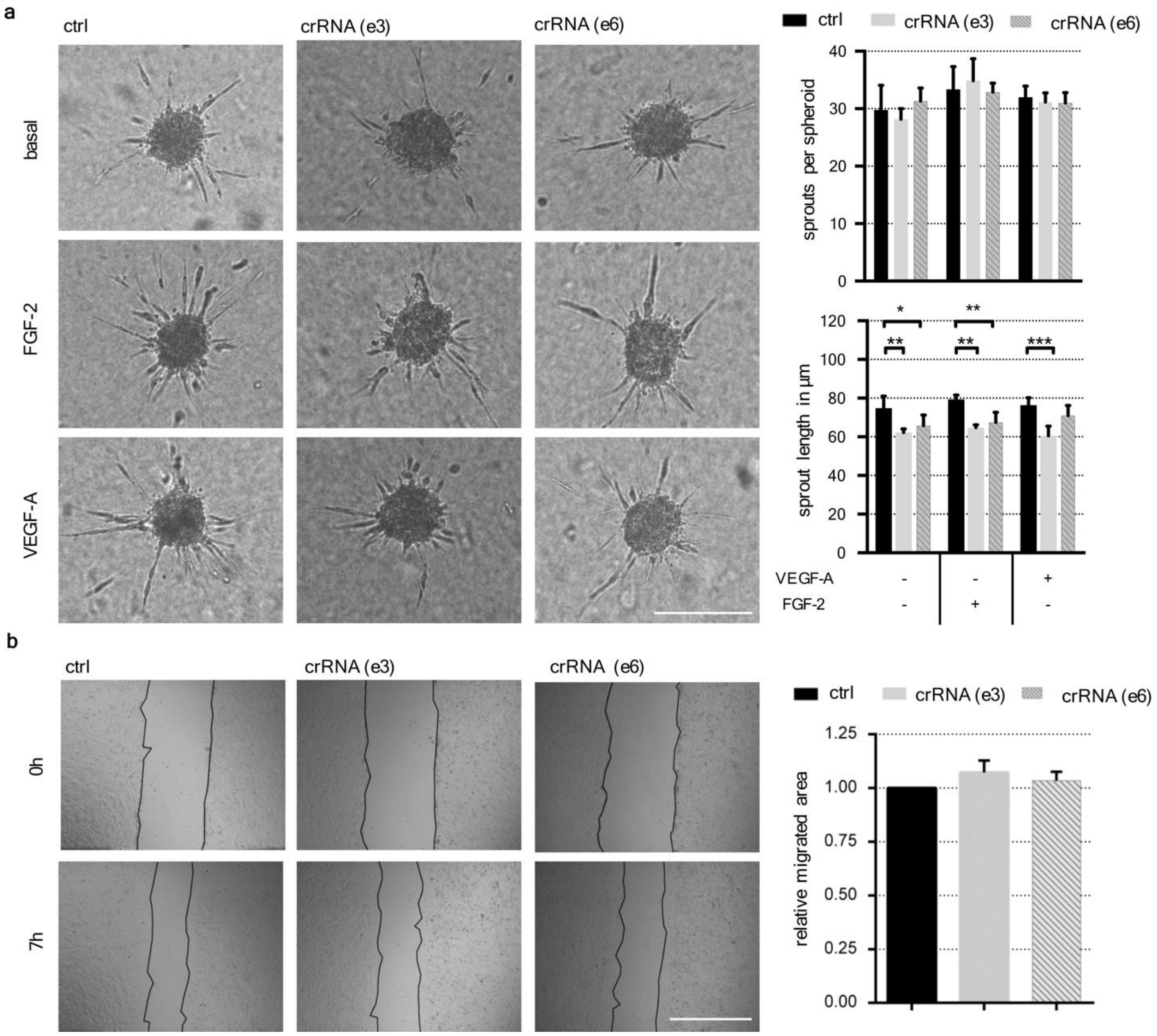

Figure S8. Reduced angiogenic response of hCMEC/D3 upon long-term CCM3 deficiency. (a) Sprout lengths were slightly reduced after crRNA:tracrRNA:Cas9 RNP transfection under basal culture conditions and stimulation with 25 ng/ml VEGF-A or FGF-2. Scale bar ≙ 200 µm. (b) No differences were observed for cell migration rates. Scale bar ≙ 1 mm. Data are presented as mean and SD (n=3). One-way or Two-way ANOVA with Dunnett's multiple comparisons test were used for statistical analysis: * $p<0.05$; ** $p<0.01$; *** $p<0.001$.

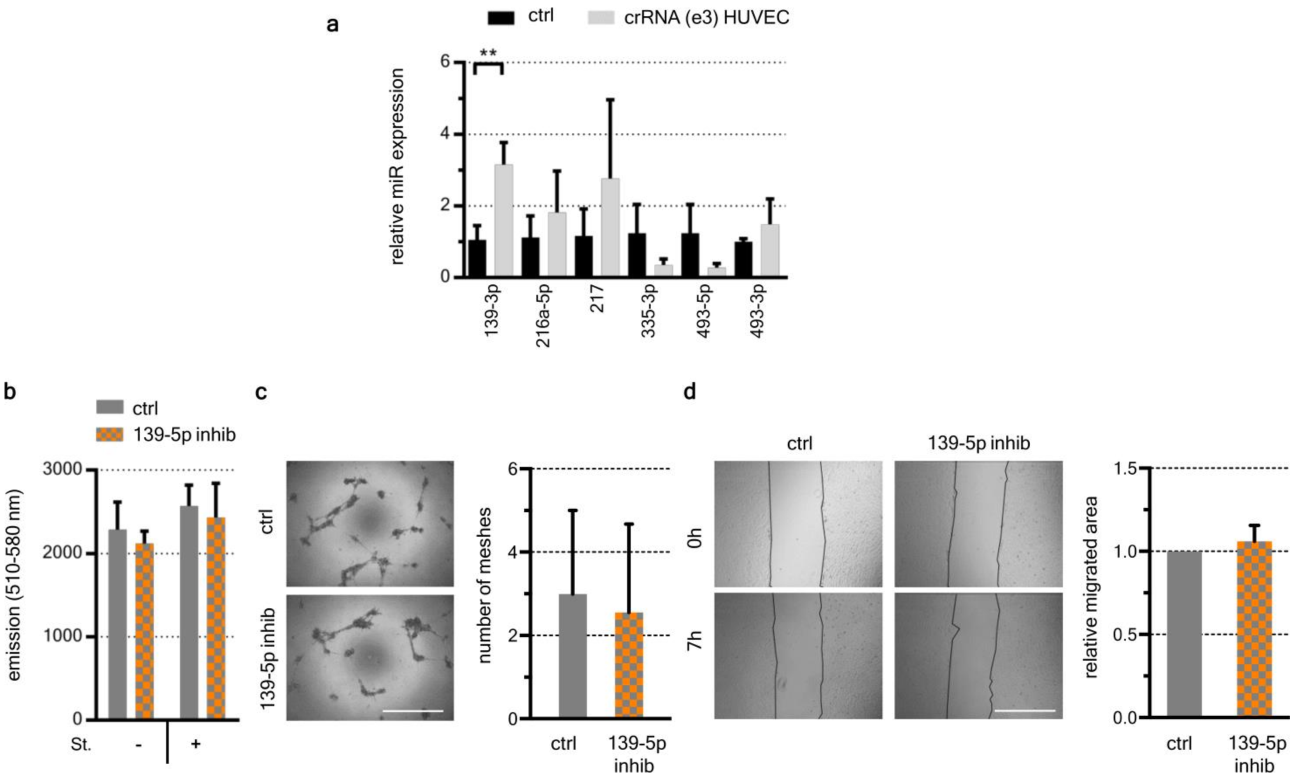

Figure S9. Validation of deregulated miRNAs and miR-139-5p inhibition in ECs. (a) Deregulated miRNAs found in *CCM3*$^{-/-}$ CI-huVECs were validated in HUVECs (passage 18) after crRNA(e3):tracrRNA:Cas9 transfection. Apoptosis (b), tube formation (c) and migration (d) were unaffected by acute miR-139-5p downregulation in *CCM3*$^{-/-}$ CI-huVECs. Scale bars ≙ 1 mm (b, c). ctrl = non-targeting miR-inhibitor, St. = Staurosporine. Data are presented as mean and SD (n=3-4). Student's t-test and One-sample t-test were used for statistical analysis.

Table S1. Potential off-target loci of crRNAs targeting *CCM3* exon 3 (crRNA e3) and exon 6 (crRNA e6). Mismatches in the crRNA binding regions are marked in red. No off-target mutations were observed by T7E1 cleavage assay in any CRISPR/Cas9 treated cell type.

crRNA	Gene	Localization	crRNA off-target sequence (5'-3')	PAM sequence	T7E1 positive		
					HUVEC (N=3)	CI-huVEC (N=7-11)	hCMEC/D3 (N=3)
crRNA e3	*ZNF256* (19q13.43)	exonic	GCTGTCACCTCATTAAACAC	CGG	0 %	0 %	0 %
	DGKH (13q14.11)	intronic	TAATTAAGCTCATTAAACAC	AGG	0 %	0 %	0 %
crRNA e6	*SLC26A2* (5q32)	exonic	TTACTCATGCCTTTTTGTTT	AGG	0 %	0 %	0 %
	RP11-283G6.4 (12p11.23)	intronic	GTGGCCATCCCTTTTCGTTT	TGG	0 %	0 %	0 %

References:

1. **Sanjana NE, Shalem O, Zhang F.** Improved vectors and genome-wide libraries for CRISPR screening. *Nat Methods*. 2014; 11: 783-4.
2. **Huang X, Zhou G, Wu W, *et al.*** Editing VEGFR2 Blocks VEGF-Induced Activation of Akt and Tube Formation. *Invest Ophthalmol Vis Sci*. 2017; 58: 1228-36.
3. **Ran FA, Hsu PD, Wright J, *et al.*** Genome engineering using the CRISPR-Cas9 system. *Nat Protoc*. 2013; 8: 2281-308.
4. **Spiegler S, Kirchmaier B, Rath M, *et al.*** *FAM222B* Is Not a Likely Novel Candidate Gene for Cerebral Cavernous Malformations. *Mol Syndromol*. 2016; 7: 144-52.
